# Diurnal Variation in Systemic Acute Inflammation and Clinical Outcomes Following Severe Blunt Trauma

**DOI:** 10.3389/fimmu.2019.02699

**Published:** 2019-11-20

**Authors:** Akram M. Zaaqoq, Rami A. Namas, Othman Abdul-Malak, Khalid Almahmoud, Derek Barclay, Jinling Yin, Ruben Zamora, Matthew R. Rosengart, Timothy R. Billiar, Yoram Vodovotz

**Affiliations:** ^1^Department of Surgery, University of Pittsburgh, Pittsburgh, PA, United States; ^2^Department of Critical Care Medicine, MedStar Washington Hospital Center, Washington, DC, United States; ^3^Center for Inflammation and Regeneration Modeling, McGowan Institute for Regenerative Medicine, University of Pittsburgh, Pittsburgh, PA, United States

**Keywords:** circadian rhythm, blunt trauma, chemokines, nervous system, acute inflammation

## Abstract

Animal studies suggest that the time of day is a determinant of the immunological response to both injury and infection. We hypothesized that due to this diurnal variation, time of injury could affect the systemic inflammatory response and outcomes post-trauma and tested this hypothesis by examining the dynamics of circulating inflammatory mediators in blunt trauma patients injured during daytime vs. nighttime. From a cohort of 472 blunt trauma survivors, two stringently matched sub-cohorts of moderately/severely injured patients [injury severity score (ISS) >20] were identified. Fifteen propensity-matched, daytime-inured (“mDay”) patients (age 43.6 ± 5.2, M/F 11/4, ISS 22.9 ± 0.7) presented during the shortest local annual period (8:00 am−5:00 pm), and 15 propensity-matched “mNight” patients (age 43 ± 4.3, M/F 11/4, ISS 24.5 ± 2.5) presented during the shortest night period (10:00 pm−5:00 am). Serial blood samples were obtained (3 samples within the first 24 h and daily from days 1–7) from all patients. Thirty-two plasma inflammatory mediators were assayed. Two-way Analysis of Variance (ANOVA) was used to compare groups. Dynamic Network Analysis (DyNA) and Dynamic Bayesian Network (DyBN) inference were utilized to infer dynamic interrelationships among inflammatory mediators. Both total hospital and intensive care unit length of stay were significantly prolonged in the mNight group. Circulating IL-17A was elevated significantly in the mNight group from 24 h to 7 days post-injury. Circulating MIP-1α, IL-7, IL-15, GM-CSF, and sST2 were elevated in the mDay group. DyNA demonstrated elevated network complexity in the mNight vs. the mDay group. DyBN suggested that cortisol and sST2 were central nodes upstream of TGF-β1, chemokines, and Th17/protective mediators in both groups, with IL-6 being an additional downstream node in the mNight group only. Our results suggest that time of injury affects clinical outcomes in severely injured patients in a manner associated with an altered systemic inflammation program, possibly implying a role for diurnal or circadian variation in the response to traumatic injury.

## Introduction

Despite advances in critical care over the past 40 years, severe blunt trauma is still associated with significant long-term morbidity and mortality (1, 2). Systemic acute inflammation is thought to be a key driver of post-injury critical illness (3). Although properly regulated inflammation is crucial for promoting adequate tissue healing and recovery, an overly exuberant or insufficient response may result in immune dysregulation as well as secondary tissue and organ damage that can be complicated by prolonged hospitalization (4–6).

The timing of the light-dark cycle results in a shift of the circadian rhythms, functioning to synchronize and coordinate organ systems in response to environmental light dynamics (7, 8). The immune system is under direct circadian control by systemic cues and molecular clocks within immune cells (9). These oscillations may also help to promote tissue recovery and the clearance of potentially harmful cellular elements from the circulation (8, 9). The suprachiasmatic nucleus (SCN) of the anterior hypothalamus contains specialized neurons that receive photo input through the retinohypothalamic tract (RHT) and non-photo cues by disparate neural inputs (9, 10). Subsequently, the nervous system regulates the inflammatory response through neuronal and neuroendocrine pathways (7).

At the molecular level, there are multiple sets of transcription factors that result in autoregulatory transcription-translation feedback loops of core clock genes, such as BMAL1 and CLOCK, which in turn control the output of circadian physiology and behavior (11). Recent studies have suggested that this complex system impacts inflammatory responses in the context of sepsis (12–14).

To address whether the time of injury alters trauma-induced systemic inflammation and, thereby, trauma-related outcomes, we retrospectively analyzed data from a large cohort of blunt trauma patients who survived up to discharge. A granular, temporal sampling of the early systemic inflammatory response of both the overall cohort as well as in propensity-matched sub-cohorts of patients injured during the day vs. the nighttime, combined with data-driven computational methods, allowed us to define differential dynamic inflammation networks as a function of time of injury. Our analyses revealed that early, persistent changes in post-injury inflammation manifest in unique biomarker patterns associated with the time of injury. Also, these patterns, which are independent of the mechanism of injury, injury severity, age, or gender suggest a diurnal, and perhaps also circadian, control of post-trauma inflammation that impacts clinical outcomes.

## Materials and Methods

The study protocol was reviewed and approved by the University of Pittsburgh Institutional Review Board (IRB), and has been detailed previously (15, 16). Written informed consent was taken from each patient or the next of kin in line with our locally agreed protocols with Institutional Review Board regulations. Patients eligible for enrollment in the study were at least 18 years of age, admitted to the intensive care unit (ICU) within 24 h of injury, and, per the treating physician, were expected to live more than 24 h. Reasons for ineligibility were isolated head injury, penetrating trauma (due to our focus on blunt trauma), and pregnancy. Blunt trauma patients were enrolled in the study from 2004 to 2012. Laboratory results and basic demographic data were recorded in the database directly from the electronic medical record. Three plasma samples, starting with the initial blood draw upon arrival to the emergency department (ED), were assayed within the first 24 h following trauma and then daily between 4:00 and 5:00 a.m. from days 1 to 7 post-injury. The blood samples were centrifuged, and plasma aliquots were stored in cryoprecipitate tubes at −80°C for subsequent analysis of inflammatory mediators.

### Study Design

**Figure 1:**
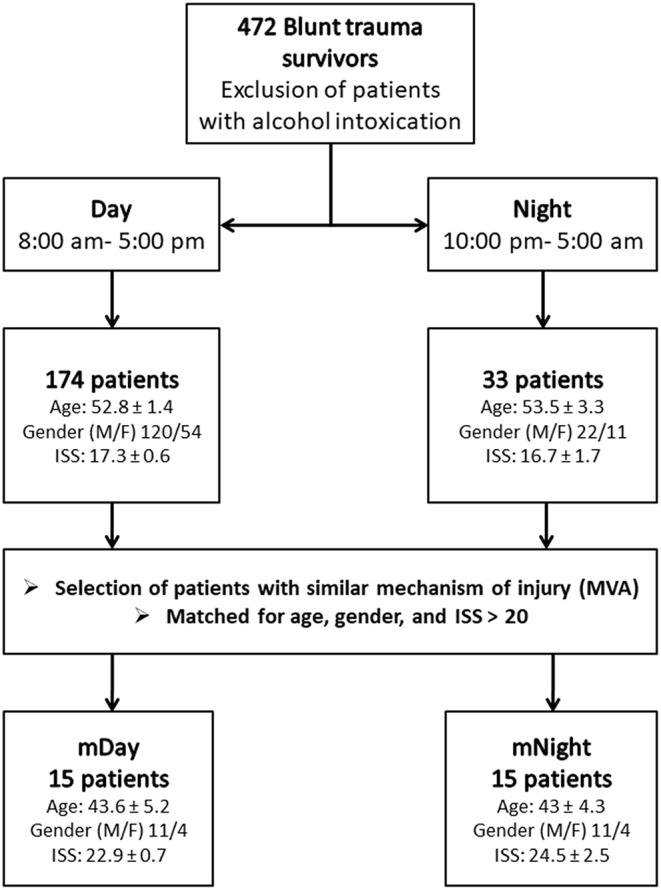
Flow chart of recruitment and study participation. Sub-cohorts were matched according to mechanisms of injury, age, gender ratio, and injury severity score (ISS) >20.

A retrospective study involving a cohort of 472 blunt trauma survivors (330 males and 142 females, age 48.4 ± 0.9, ISS 19.6 ± 0.5) who were admitted to the emergency department of the Presbyterian University Hospital (Level 1 trauma center) (16) (Figure 1). Exclusion criteria included patients with evidence of alcohol intoxication upon admission due to the potential impact of alcohol on systemic inflammation. From this cohort, “Day” patients were identified based on the time of presentation to Presbyterian University Hospital during the shortest daylight in Pittsburgh, PA, USA throughout the year (8:00 a.m.−5:00 p.m.) and “Night” patients presented during the shortest night period (10:00 p.m.−5:00 a.m.) (Figure 1). The overall demographics, mechanism of injury, clinical data, and co-morbidities of the 174 (121 males and 53 females, age 52.8 ± 1.4, ISS 17.3 ± 0.6) patients in the Day group vs. 33 (22 males and 11 females, age 53.3 ± 3.3, ISS 16.7 ± 1.7) patients in the Night group are shown in Table 1. Initially, we sought to avoid the confounding impact related to the type of mechanism of injury by selecting patients based on the predominant mechanism of injury in both cohorts, i.e., motor vehicle accidents (MVA). Given that the Night group exhibited statistically significantly higher rates of head and extremity injuries when compared to the Day group (see Table 1) as revealed by the abbreviated injury scale (AIS), we next performed a one-to-one propensity matching based on age, sex, and ISS > 20, which yielded two sub-cohorts of 15 Day patients (mDay) matched to 15 Night patients (mNight) (Figure 1).

**Table 1:** Demographics, mechanism of injury, co-morbid conditions, and clinical outcomes of the total trauma cohort compared to the Day and Night cohorts.

**Variables**	**Total cohort** ***n* = 472**	**Day cohort** ***n =* 174**	**Night cohort** ***n =* 33**	***P***
**DEMOGRAPHICS**				
Age, yr	48.4 ± 0.9	52.8 ± 1.4	53.5 ± 3.3	0.9
Sex, male/female	330/142	120/54	22/11	0.8
Injury severity score (ISS)	19.6 ± 0.5	17.3 ± 0.6	16.7 ± 1.7	0.5
**ABBREVIATED INJURY SCALE (AIS)**				
Head and Neck	1.4 ± 0.08	0.89 ± 0.1	1.69 ± 0.3	0.003
Face	0.39 ± 0.04	0.31 ± 0.05	0.27 ± 0.1	0.9
Chest	2.02 ± 0.07	1.95 ± 0.1	2.06 ± 0.3	0.7
Abdomen	1.16 ± 0.06	1.04 ± 0.09	0.94 ± 0.2	0.6
Extremities	1.5 ± 0.06	1.5 ± 0.09	1 ± 0.2	0.017
External	0.67 ± 0.02	0.66 ± 0.04	0.61 ± 0.09	0.6
**MECHANISM OF INJURY**				
Motor vehicle accident (MVA), *n* (%)	269 (57%)	106 (60.9%)	19 (57.5)	0.7
Fall, *n* (%)	102 (21.6%)	36 (20.7%)	10 (30.3%)	0.2
Motorcycle, *n* (%)	65 (13.8%)	20 (11.5%)	2 (6.1%)	0.4
Other, *n* (%)	36 (7.6%)	12 (6.9%)	2 (6.1%)	0.9
**CO-MORBID CONDITIONS**				
Hypertension, *n* (%)	143 (30.3%)	68 (39.1%)	8 (24.2%)	0.1
Diabetes, *n* (%)	58 (12.3%)	29 (16.7%)	5 (15.2%)	0.8
Psychiatric conditions, *n* (%)	58 (12.3%)	27 (15.5%)	5 (15.2%)	0.9
Thyroid diseases, *n* (%)	26 (5.5%)	12 (6.9%)	4 (12.1%)	0.3
Bronchial asthma, *n* (%)	28 (5.9%)	28 (16.1%)	4 (12.1%)	0.8
None, *n* (%)	154 (32.6%)	55 (31.6%)	12 (36.4%)	0.8
**OUTCOME**				
Mechanical ventilation, days	3.1 ± 0.3	2.7 ± 0.5	2.2 ± 0.7	0.7
Intensive Care Unit length of stay, days	7.01 ± 0.36	5.9 ± 0.5	6.7 ± 1.3	0.8
Total hospital length of stay, days	12.72 ± 0.44	11.6 ± 0.7	12.1 ± 1.7	0.7

*Values are expressed as mean ± SEM. One-Way ANOVA or Fisher exact test were used as appropriate with statistical significance set at P < 0.05*.

### Clinical Data Collection

Clinical data, including injury severity score (ISS), abbreviated injury scale (AIS) score, Marshall Multiple Organ Dysfunction (MOD) score, ICU LOS, hospital LOS, and days on mechanical ventilation were collected from the hospital inpatient electronic and trauma registry database. ISS (17) and AIS scores (18) were calculated for each patient by a single trauma surgeon after attending radiology evaluations were finalized. The ISS is based on an anatomical scoring system that provides an overall score for patients with multiple injuries (17). Each injury is assigned an AIS score, allocated to one of six body regions: head, face, chest, abdomen, extremities (including pelvis), and external. We focused our studies on trauma patients with ISS > 20, which would be considered moderate/severe (19).

As an index of organ dysfunction, the MOD score (20) (ranging from 0 to 24) was calculated. In brief, six variables were obtained from the electronic trauma data registry including (a) the respiratory system (PO_2_/FIO_2_ ratio); (b) the renal system (serum creatinine concentration); (c) the hepatic system (serum bilirubin concentration); (d) the hematologic system (platelet count); (e) the central nervous system (Glasgow Coma Scale); and (f) the cardiovascular system- the pressure-adjusted heart rate (PAR).

### Analysis of Inflammatory Mediators

Blood samples were collected into citrated tubes via indwelling catheters within 24 h of admission and daily for 7 days post-injury. The blood samples were centrifuged, and plasma aliquots were stored in cryoprecipitate tubes at −80°C for subsequent analysis of inflammatory mediators. The human inflammatory MILLIPLEX™ MAP Human Cytokine/Chemokine Panel-Premixed 26 Plex (Millipore Corporation, Billerica, MA) and Luminex™ 100 IS (Luminex, Austin, TX) was used to measure plasma levels of interleukin (IL)-1β, IL-1 receptor antagonist (IL-1Ra), IL-2, soluble IL-2 receptor-α (sIL-2Rα), IL-4, IL-5, IL-6, IL-7, IL-8, IL-10, IL-13, IL-15, IL-17A, interferon (IFN)-γ, IFN-γ inducible protein (IP)-10 (CXCL10), monokine induced by gamma interferon (MIG; CXCL9), macrophage inflammatory protein (MIP)-1α (CCL3), MIP-1β (CCL4), monocyte chemotactic protein (MCP)-1 (CCL2), granulocyte-macrophage colony stimulating factor (GM-CSF), Eotaxin, and tumor necrosis factor-alpha (TNF-α). The human Th17 MILLIPLEX Panel kit (Millipore Corporation, Billerica, MA) was used to measure IL-9, IL-21, IL-22, IL-23, IL-17E/25, and IL-33. The Luminex™ system was used in accordance to the manufacturer's instructions. Soluble ST2 (sST2) was measured by a sandwich ELISA assay (R&D Systems, Minneapolis, MN). ${\text{NO}}_{2}^{-}$/${\text{NO}}_{3}^{-}$ was measured using the nitrate reductase/Griess assay (Cayman Chemical Co., Ann Arbor, MI). Serum cortisol and transforming growth factor (TGF)-β1 were assayed using commercially available enzyme-linked immunosorbent assays (ELISA) kits (R&D Systems, Minneapolis, MN) according to the manufacturer's instructions. In brief, active vs. latent TGF-β1 were assayed as follows: To collect platelet-poor plasma, plasma was collected on ice using EDTA as an anticoagulant and centrifuged for 15 min at 1,000 × g within 30 min of collection followed by an additional centrifugation step of the plasma at 10,000 × g for 10 min at 2–8°C for complete platelet removal. To activate latent TGF-β1 to immunoreactive TGF-β1 detectable by the Quantikine® TGF-β1 immunoassay, 20 μL of 1 N HCl was added to each 40 μL of plasma, mixed and incubated for 10 min at room temperature. Next, the acidified samples were neutralized by adding 20 μL of 1.2 N NaOH/0.5 M HEPES, mixed and then diluted with calibrator diluent prior to the assay. To perform the assay, 50 μL of diluent RD1-73 were added to each well-followed by addition of 50 μL of recombinant human TGF-β1 standard, control, or activated sample per well and incubated for 2 h at room temperature. Following 4 aspirations/wash steps, 100 μL of TGF-β1 conjugate was added to each well and incubated for an additional 2 h at room temperature, washed, and then 100 μL substrate solution was added to each well and incubated for 30 min at room temperature. Finally, 100 μL of stop solution was added to each well and the optical density (wavelength set at 450 nm) was determined.

### Statistical Analysis

All data are expressed as mean ± SEM. Group-time interaction of plasma inflammatory mediators' levels was determined by Two-way analysis of variance (ANOVA) which was confirmed by non-parametric Mann-Whitney U test to compare the *P*-values generated by the Two-way ANOVA in the case where values were not normally distributed, all using SigmaPlot™ 11 software (Systat Software, Inc., San Jose, CA). Fisher's exact test was performed for categorical data using Graphpad PRISM (GraphPad Software, Inc., La Jolla, CA). The correlation between different inflammatory mediators was determined by Spearman's correlation using the actual values of these inflammatory mediators. *P* < 0.05 was considered statistically significant for all analyses.

### Dynamic Bayesian Network Inference

Dynamic Bayesian Network (DyBN) inference was carried out to define the most likely single-network structure that best characterizes the dynamic interactions among systemic inflammatory mediators across time, in the process suggesting likely feedback structures that define central nodes. The networks might also suggest possible mechanisms by which the progression of the inflammatory response differs within a given experimental group. This analysis was carried out using MATLAB™ (The MathWorks, Inc., Natick, MA), using an algorithm adapted from Grzegorczyk and Husmeier (21) and revised by our group (22). In this analysis, inflammatory mediators were represented at multiple time points within the same network structure. In this approach, time was modeled discretely as in a discrete Markov chain. Each mediator was given a time index subscript indicating the time slice to which it belonged. Additional temporal dependencies were represented in a DyBN by edges between time slices. Each node in the network was associated with a conditional probability distribution of a variable that is conditioned upon its parents (upstream nodes). This particular network structure was used to assess the dominant inflammatory mediators and the probable interaction among various mediators, including possible feedback (21).

### Data-Driven Modeling: Dynamic Network Analysis (DyNA)

DyNA was carried out as described previously (15, 23, 24). The goal of this analysis was to gain insights into dynamic changes in network connectivity of the post-traumatic inflammatory response for both day and night over time. The mathematical formation of this method is essential to calculate the correlation between the variables by which we can examine their dependence. To do so, inflammatory mediator networks were created in adjacent 8-h time periods (0–8, 8–16, and 16–24 h) using MATLAB® (The MathWorks, Inc., Natick, MA). Connections in the network were created if the correlation coefficient between two nodes (inflammatory mediators) was greater or equal to a threshold of 0.7. For the network density calculation, to account for network sizes (number of significantly altered nodes) in the adjacent 8-h time periods detailed above, we utilized the following formula: [a minor revision of the one reported by Assenov et al. (25)].

$$\begin{array}{l} \frac{\text{Total number of edges }*\text{ Number of total nodes}}{\text{Maximum possible edges among total nodes}} \end{array}$$

## Results

### Overview of Demographics and Outcomes in a Large Cohort of Blunt Trauma Patients

Over the 8-year study period, 472 blunt trauma patients admitted to the ICU were enrolled in the study, as previously described (15, 16). The majority of the 472 trauma patients were males (70.6 %), with a mean age of 48.4 ± 0.9 years and a mean ISS of 19.6 ± 0.5. These patients sustained blunt trauma in the form of MVA (57%), falls (21.6%), motorcycle accidents (13.8%), and others (7.6%). The AIS analysis revealed that the Night cohort exhibited statistically significantly higher rates of head (1.69 ± 0.3 vs. 0.89 ± 0.1; *P* < 0.003) and extremity (1 ± 0.2 vs. 1.5 ± 0.09; *P* = 0.017) injuries when compared to the day group (Table 1). The average ICU LOS was 7.01 ± 0.36 d, the mean hospital LOS was 12.7 ± 0.4 d, and the mean number of days on a mechanical ventilator was 3.1 ± 0.3 d.

### Characteristics of Day and Night Injury Cohorts: Demographics, Outcomes, and Propensity Matching

A total of 174 patients met our definition of being injured during the day and 33 patients injured during the night as defined in the *Materials and Methods* (Table 1). Overall, males were predominant in both the Day and Night cohorts (68.9 and 66.7%, respectively), with no statistical difference in mean age (52.8 ± 1.4 vs. 53.5 ± 3.3; *P* = 0.9) between the two cohorts. Also, there was no statistically significant difference in ISS (17.3 ± 0.6 vs. 16.7 ± 1.7; *P* = 0.5), ICU LOS (5.9 ± 0.5 vs. 6.7 ± 1.3; *P* = 0.8), hospital LOS (11.6 ± 0.7 vs. 12.1 ± 1.7; *P* = 0.7), days on mechanical ventilation (2.7 ± 0.5 vs. 2.2 ± 0.7; *P* = 0.7), the prevalence of nosocomial infection (NI: 39/173 [22.5%] vs. 8/33 [24.2%]; *P* = 0.8), or the average Marshall MOD score across days 1 to 7 (1.3 vs. 1.6; *P* = 0.9) between the two cohorts.

**Figure 2:**
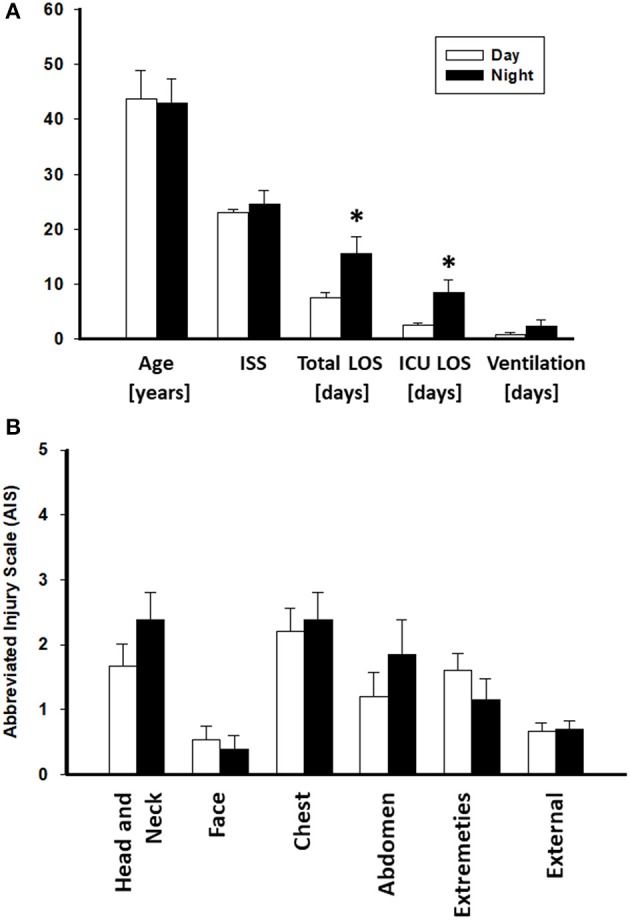
Abbreviated Injury Scale (AIS) values of the Day (*n* = 174) and Night (*n* = 33) cohorts. No statistically significant difference was found between both groups. ^*^P < 0.05 by Student's *t*-test.

A total of 15 patients in the Night cohort (matched Night [mNight]) were matched with 15 patients in Day cohort (matched Day [mDay]), according to age, gender, and ISS >20. Overall, males were predominant in both mDay and mNight sub-cohorts (73.3% in both sub-cohorts), with no statistical difference in mean age (43.6 ± 5.2 vs. 43 ± 4.3; *P* = 0.9; respectively) between the two sub-cohorts. Moreover, ISS was not statistically significantly different between both groups (22.9 ± 0.7 vs. 24.5 ± 2.5; *P* = 0.9; respectively; Figure 2A). Importantly, there were no statistically significant differences in any of the body regions between both sub-cohorts based on AIS body regions (the components of the ISS; *see Materials and Methods*) (Figure 2B).

### Greater Requirement for Surgical Interventions Needed for Trauma Management, as Well as Worse Clinical Outcomes, in Stringently Matched Night vs. Day Sub-cohorts

The mNight group required more surgical interventions than the mDay group. These interventions were mainly either reductions/fixations for simple or compound fractures: 5/15 (33.3%) of the mNight patients had orthopedic intervention vs. 2/15 (13.3%) in the mDay sub-cohort (*P* = 0.02). There was no difference in the rate of exploratory laparotomies between the sub-cohorts. In addition, there was no statistically significant difference in the rates of NI (5/15 [33.3%] vs. 2/15 [13.3%]; *P* = 0.2) and average Marshall MOD score (1.3 vs. 0.9; *P* = 0.4) between the two sub-cohorts. Interestingly, though there was no statistically significant difference in the requirement for mechanical ventilation (0.8 ± 0.3, 2.3 ± 1.1; *P* = 0.9), there was a statistically significantly longer ICU LOS (2.6 ± 0.3 vs. 8.5 ± 2.3; *P* = 0.04) and total hospital LOS (7.6 ± 0.9 vs. 15.5 ± 3; *P* = 0.02) in mNight sub-cohort when compared to the mDay group (Table 2).

**Table 2:** Demographics, mechanism of injury, co-morbid conditions, and clinical outcomes of the stringently matched (m)Day and mNight sub-cohorts.

**Variables**	**mDay** ***n =* 15**	**mNight** ***n =* 15**	***P***
**DEMOGRAPHICS**			
Age, yr	43.6 ± 5.2	43 ± 4.3	0.9
Sex, male/female	11/4	11/4	0.9
Injury severity score (ISS)	22.9 ± 0.7	24.5 ± 2.5	0.9
**MECHANISM OF INJURY**			
Motor vehicle accident (MVA), *n* (%)	11 (73.3%)	12 (80%)	0.7
Motorcycle, *n* (%)	3 (20%)	1 (6.7%)	0.3
Other, *n* (%)	1 (6.7%)	2 (13.3%)	0.5
**ABBREVIATED INJURY SCALE (AIS)**			
Head and Neck	1.3 ± 1.6	2.4 ± 1.5	0.09
Face	0.5 ± 0.8	0.4 ± 0.8	0.6
Chest	2.2 ± 1.4	2.4 ± 1.5	0.7
Abdomen	0.7 ± 1	1.8 ± 1.9	0.14
Extremities	1.6 ± 1	1.2 ± 1.1	0.3
External	0.67 ± 0.5	0.69 ± 0.5	0.9
**CO-MORBID CONDITIONS**			
Hypertension, *n* (%)	5 (33.3%)	3 (20%)	0.4
Diabetes, *n* (%)	4 (26.7%)	1 (6.7%)	0.14
Psychiatric conditions, *n* (%)	2 (13.3%)	3 (20%)	0.6
Thyroid diseases, *n* (%)	1 (6.7%)	1 (6.7%)	1
Bronchial asthma, *n* (%)	2 (13.3%)	1 (6.7%)	0.5
None, *n* (%)	6 (40%)	8 (53.3%)	0.5
**OUTCOME**			
Mechanical ventilation, days	0.8 ± 0.3	2.3 ± 1.1	0.9
Intensive Care Unit length of stay, days	2.6 ± 0.3	8.5 ± 2.3	0.043
Total hospital length of stay, days	7.6 ± 0.9	15.5 ± 3	0.02

*Values are expressed as mean ± SEM. One-Way ANOVA or Fisher exact test were used as appropriate with statistical significance set at P < 0.05*.

### Divergent Systemic Inflammatory Responses in Stringently Matched Day vs. Night vs. Sub-cohorts

**Figure 3:**
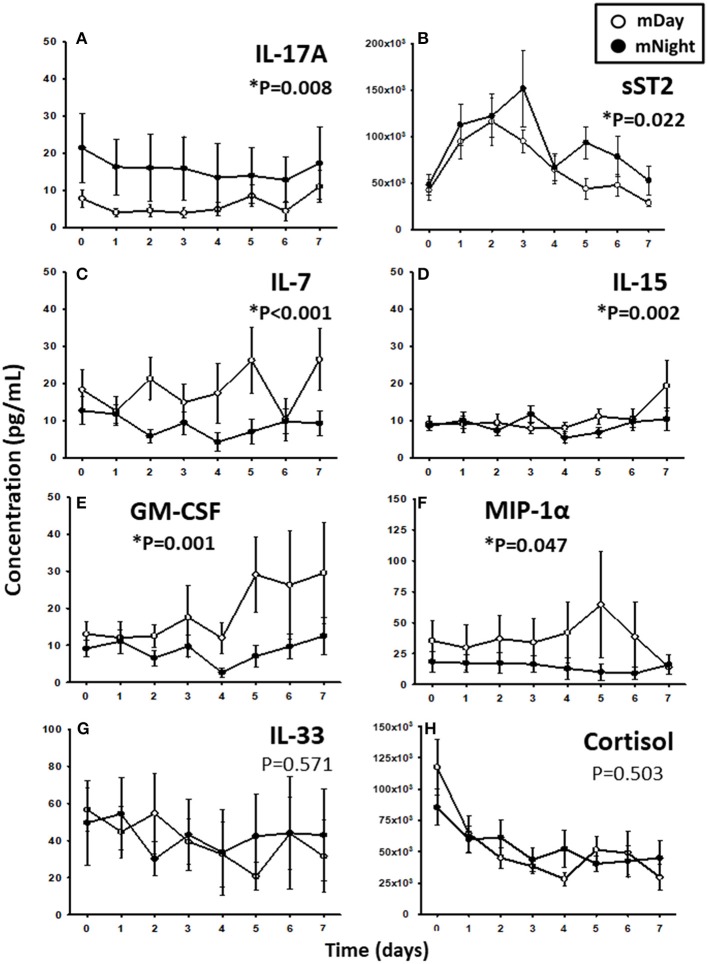
Time course analysis of inflammatory mediators in the matched (m)Day sub-cohort (*n* = 15) vs. the mNight sub-cohort (*n* = 15). **(A)** Time course of IL-17A. **(B)** Time course of sST2. **(C)** Time course of IL-7. **(D)** Time course of IL-15. **(E)** Time course of GM-CSF. **(F)** Time course of MIP-1α. **(G)** Time course of IL-33. **(H)** Time course of cortisol. The indicated inflammatory mediators were assessed in serial plasma samples obtained at the indicated time points. Values are mean ± SEM (pg/mL). ^*^P < 0.05 by Two-Way ANOVA (also indicated in bold).

**Figure 4:**
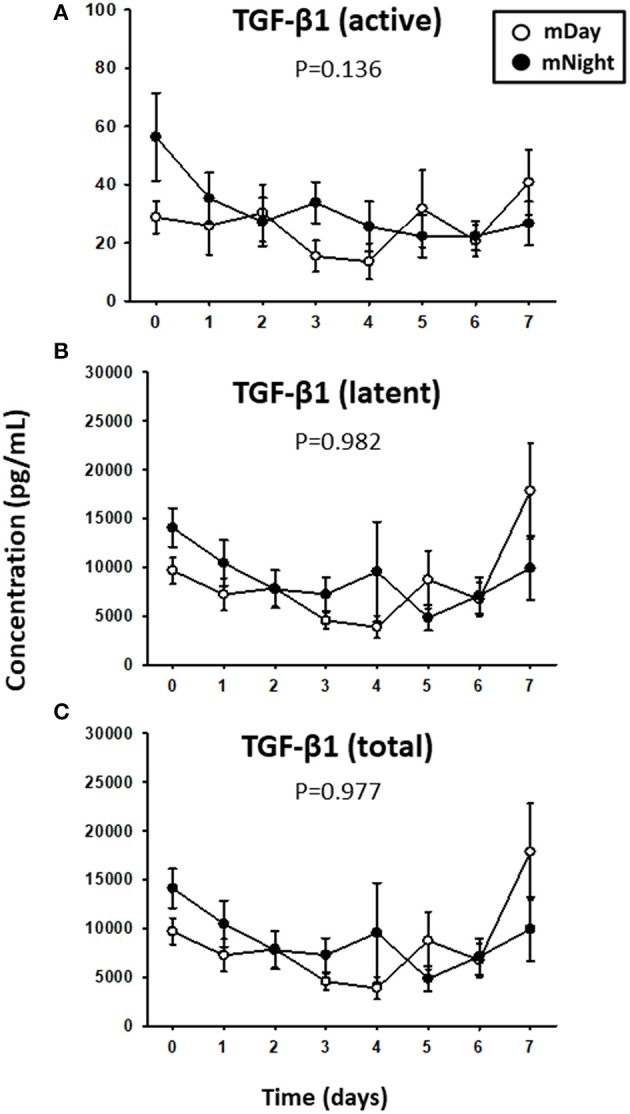
Time course analysis of active, latent, and total TGF-β1 in the matched (m)Day group (*n* = 15) vs. the mNight group (*n* = 15). **(A)** Time course of active TGF-β1. **(B)** Time course of latent TGF-β1. **(C)** Time course of total (latent + active) TGF-β1. Mean circulating levels of TGF-β1 in both the mDay (*n* = 15) and mNight (*n* = 15) sub-cohorts. The indicated inflammatory mediators were assessed in serial plasma samples obtained at the indicated time points. Values are mean ± SEM (pg/ml). None of the levels were significantly different between mDay and mNight patients.

Since trauma and subsequent organ dysfunction elicit a systemic inflammatory response which is regulated in part by diurnal and circadian rhythms, we hypothesized that the dynamics of inflammatory mediators could differ according to the time of injury and day/night cycle. We observed that IL-17A (*P* = 0.008; Figure 3A) and sST2 (*P* = 0.022; Figure 3B) were significantly higher in the mNight sub-cohort compared to the mDay sub-cohort. In contrast, IL-7 (*P* < 0.001; Figure 3C), IL-15 (*P* = 0.002; Figure 3D), GM-CSF (*P* = 0.001; Figure 3E), and MIP-1α (*P* = 0.047; Figure 3F) were significantly lower in the mNight sub-cohort. Notably, the circulating levels of the ligand for ST-2, IL-33 (26, 27), which we previously reported as being elevated in trauma patients (28), were not significantly different in the mDay vs. mNight cohorts (Figure 3G). Though cortisol is a key mediator whose levels vary with time of day in healthy individuals (29, 30), no differences in circulating cortisol levels were observed between mDay and mNight patients (Figure 3H). Also, there was no statistically significant difference in active, latent, and total TGF-β1 (Figure 4) between the mDay and mNight sub-cohorts.

### Different Dynamic Inflammatory Networks Inferred in Day vs. Night Patients

**Figure 5:**
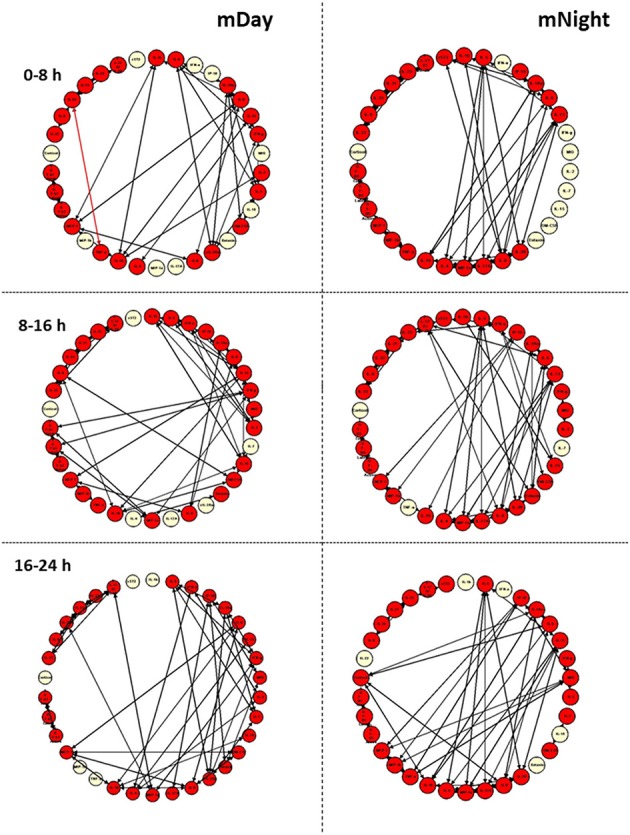
Dynamic network analysis (DyNA) of inflammatory mediators in the matched (m)Day and mNight sub-cohorts (*n* = 15 each). DyNA was carried out using data on all inflammatory mediators assessed, as described in the *Materials and Methods*. This analysis suggested a higher dynamic network complexity/connectivity in the mNight as compared to the mDay group.

We next sought to gain insights into the systemic inflammatory programs present in mDay and mNight sub-cohorts by examining dynamic network connectivity among inflammatory mediators using Dynamic Network Analysis (DyNA) (15, 23, 24). In addition to determining which networks were present at specific time intervals, we also assessed the total degree of connectivity at each of these intervals. Figure 5 shows the detailed DyNA results for Day and Night in three different time periods following presentation (0–8, 8–16, and 16–24 h). We focused especially on DyNA connectivity among nodes with ≥ 4 connections. In the mDay group, these highly connected sub-networks initially involved MCP-1/IL-10/sIL-2Rα/IL-5/IL-7/IFN-γ/IL-6, and IL-1RA (0–8 h), and then MCP-1/IL-1β/IL-2/IL-5/IL-13/IL-10/IL-15/IFN-γ over the period of 8–16 h (Figure 5).

**Figure 6:**
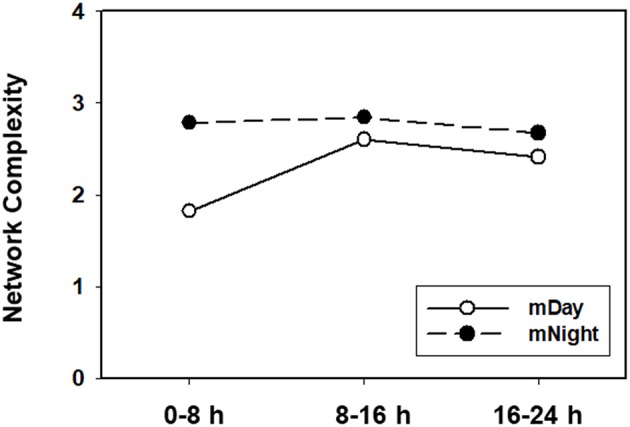
Quantification of dynamic network complexity in the mDay and mNight sub-cohorts. The mNight group exhibited relatively a higher network density at 0–8 h time point which decreased over time but remained higher than the mDay group.

In contrast, network analysis of the mNight sub-cohort data revealed interactions among IL-4/IL-5/IL-6/IL-8/IL-13/IL-17A/sIL-2Rα/IL-1Rα/MIP-1α over the first 0–8 h. During the 8–16 h time period, the network interactions included sIL-1Rα/IL-8/IL-6/Eotaxin and IL-10/IP-10/MCP-1/MIP-1β. The overall degree of network complexity was lower in the mDay group over the first 0–8 h period, and then increased over time approaching the degree of inflammatory network complexity of mNight patients by 16–24 h (Figure 6).

**Figure 7:**
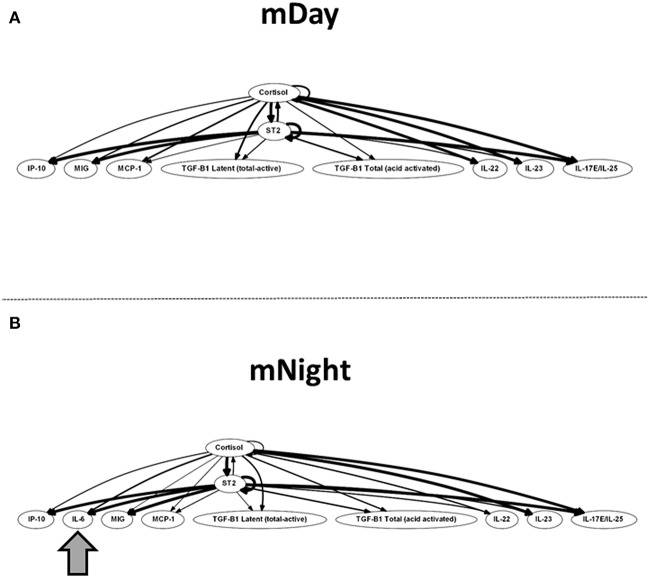
Dynamic Bayesian Network (DyBN) of inflammation biomarkers in matched **(A)** (m)Day and **(B)** mNight sub-cohorts. DyBN suggested that both cortisol and sST2 affects the production of MIG/CXCL9, IP-10, MCP-1, TGF-β1 latent, TGF-β1 total, IL-22, IL-23, and IL-17E/IL-25 production in the first 24 h post-injury in both groups.

Finally, we sought to define potential feedback structures in the dynamic networks of inflammation associated with the first 24 h post-injury during the day vs. night, and to this end, we employed DyBN inference as in previous studies (31, 32). Based on this analysis, we inferred a central motif involving dynamic interactions between cortisol and sST2 in both sub-cohorts, affecting the levels of MIG/CXCL9, IP-10/CXCL10, MCP-1/CCL2, TGF-β1 latent, TGF-β1 total, IL-22, IL-23, and IL-17E/IL-25 (Figure 7). Notably, the only differentiating feature in these networks was the presence of IL-6 as a downstream node in mNight but not mDay patients.

## Discussion

The interactions of earth's rotation, sleep-wake cycle, the individual suprachiasmatic nucleus of the hypothalamus, and cellular core clock genes led to the evolution of daily circadian rhythms. Numerous physiological systems are under that circadian control, including inflammatory and immune responses (8, 33–35). Previous animal studies confirmed that murine responses to various pathogens as well as pro-inflammatory cytokines are under circadian control (36–40). Moreover, a recent study demonstrated that circadian rhythm was disrupted in trauma patients who went on to become septic (41). However, our study is the first to test directly for an association between diurnal variability and blunt trauma-induced clinical outcomes and systemic inflammatory responses in human subjects. In this study, we found worse clinical outcomes and more complex dynamic networks of systemic inflammation in trauma patients injured during the night as compared to during the day.

Trauma triggers a robust inflammatory response, which is important for an effective resolution of injury (16, 24, 42). However, the dysregulated immune response can impair recovery and complicate the clinical outcome (43, 44). In parallel, key components of the immune system exhibit circadian rhythmicity, which is dependent on the rest-activity diurnal phase (45). Indeed, diurnal variation was demonstrated previously in circulating levels of human peripheral blood mononuclear cell (PBMC) subsets and serum cytokine and cytokine receptors including IL-2, IL-10, GM-CSF, IL-1β, IL-6, TNF-α, MCP-1/JE, CCR2/CD192, IFN-γ, and IFN receptors (46–48). Thus, we hypothesized that the timing of the insult relative to the circadian oscillation of the immune system would trigger differential immune/inflammatory response associated with divergent clinical outcomes.

In the present study, we focused on the potential role of time of injury in propensity-matched, moderately/severely injured patients. A key observation was the association between nighttime injury and adverse clinical outcomes, namely longer intensive care unit and hospital length of stay. While controversial factors related to the quality of medical care during nighttime (49–51) or other variables such as hospital volume and socioeconomic factors cannot be ruled out in our study, our data suggest that patients injured during the night require additional and more extensive procedures rather than experiencing worse outcomes with the same degree of intervention. Moreover, the use of stringent propensity matching further reduces the likelihood of artifactual reasons underlying our findings.

Our dynamic network analyses suggest potential mechanisms underlying the differential inflammatory responses in daytime- vs. nighttime-injured patients. While neither cortisol levels nor levels of TGF-β1 and several other mediators were statistically significantly different in mDay vs. mNight, several other pathways were inferred to be activated differentially in patients injured at night vs. during the day. The lack of statistically significant differences in circulating cortisol levels in mNight vs. mDay patients suggests that trauma induces derangements in the known diurnal variation of this mediator. Indeed, cortisol dysregulation and the role of adrenal insufficiency have been appreciated as key aspects of trauma-induced critical illness (52, 53). Importantly, cortisol is a key regulator of systemic inflammation, and we observed differences in circulating levels of multiple cytokines and chemokines. Within the panoply of inflammatory and immune pathways known to be by a traumatic injury (54, 55), there has been a recent focus on type 17 immunity (56). Notably, circulating IL-17A levels were higher in mNight patients as compared to mDay patients, suggesting the possibility that a dysregulated IL-17A response may in part underlie the worse clinical outcomes in mNight patients. Moreover, circadian rhythms are known to impact Th17 development and function (57). We have previously utilized correlation analyses (IL-17A vs. GM-CSF and IL-17A vs. IL-10 to suggest the presence of pathogenic and non-pathogenic Th17 cells) (15). In a similar analysis of the mDay vs. mNight cohorts, no significant correlations were observed (Supplementary Figure 1). However, there was a trend suggesting the possible prevalence of non-pathogenic Th17 cells in the mDay sub-cohort (*r* = 0.19, *P* = 0.06; Supplementary Figure 1B) and a similar trend suggesting the prevalence of pathogenic Th17 cells in the mNight sub-cohort (*r* = 0.19, *P* = 0.051; Supplementary Figure 1C). Further studies with larger cohorts may thus be warranted to define the role, if any, of circadian alterations on Th17 (and other IL-17A-producing) cells.

We gleaned additional information by examining the dynamic evolution of networks of systemic inflammation using two different algorithms: DyNA (23) and DyBN (22). DyNA suggested a generally higher degree of inflammatory activation in mNight patients as compared to mDay patients suggesting an early activation of immune pathways compared to the mDay group. Moreover, the inflammatory responses of mDay patients were more “Th2-like” (involving IL-5, IL-10, and IL-13 early on), whereas the networks of mNight patients involved both Th2 responses (involving IL-4, IL-5, and IL-13) as well as Th17 responses (IL-17A and also IL-6, though the latter is involved in multiple other pathways). As discussed above, we speculate that the presence of type 17 immunity in the mNight group may indicate the presence of overly exuberant inflammation. We further speculate that the presence of Th2 responses in both mDay and mNight patients may underlie the absence of significant differences in nosocomial infections in patients injured at night vs. the day, though this may be a function of the limited data set in propensity-matched patients (see below).

We recognize that there are several limitations to the current study. First, this is a single-center study and thus may not be generalizable to other centers that adopt alternative management practices or challenged by different demographics or injury characteristics. These issues warrant multi-centric studies to validate the results suggested by the current one. Moreover, the number of inflammatory biomarkers analyzed was limited to the number of analytes we could measure using commercially available Luminex™ bead sets. Further future studies examining a larger panel of inflammatory biomarkers are suggested. Finally, we note that DyNA lacks mechanistic insight; however, it can be used to understand abstract key features and interactions of the trauma-induced inflammatory response.

In conclusion, we report for the first time on a potential impact of time of injury on blunt trauma outcomes and the dynamics of systemic inflammation in humans. Our findings may have larger implications for a growing body of evidence implicating the need to consider the time of day when designing therapeutic approaches, especially in the context of diseases that are strongly impacted by immune/inflammatory interactions (12, 58–60). The present study adds to this field by demonstrating that clinical outcomes of trauma patients are likewise impacted by time of injury along with other factors such as injury severity, genotype, and the character of systemic inflammation and immune dysregulation that ensues following severe injury. Given the complexity of the intertwined inflammatory, immune, and physiologic interactions in the context of circadian rhythms, it is likely that systems and computational biology approaches (59, 61) will be necessary to help define novel therapeutic control points in the context of traumatic injury (54).

## Data Availability Statement

All datasets generated for this study are included in the article/Supplementary Material.

## Ethics Statement

The studies involving human participants were reviewed and approved by University of Pittsburgh Institutional Review Board (IRB). The patients/participants provided their written informed consent to participate in this study.

## Author's Note

This work was presented in part at the 37th Annual Conference on Shock.

## Author Contributions

AZ and RN participated in study design, data collection and interpretation, and writing. OA-M and KA participated in data collection. DB and JY participated in analysis of inflammatory mediators. RZ participated in computational and statistical analysis, data interpretation, and writing. MR and TB participated in data interpretation and writing. YV participated in study design, data interpretation, and writing.

### Conflict of Interest

The authors declare that the research was conducted in the absence of any commercial or financial relationships that could be construed as a potential conflict of interest.

## Supplementary Material

Supplementary Figure 1 Correlation analyses aimed at defining Th17 cell subsets computationally. Spearman correlations were carried out on mDay vs. mNight patient cytokine data to suggest the presence of pathogenic (IL-17A vs. GM-CSF; **A,C**) and non-pathogenic (IL-17A vs. IL-10; **B,D**) Th17 cells. This analysis suggested the possible predominance of pathogenic Th17 cells in the mNight group and non-pathogenic Th17 cells in the mDay.
