# Supplementary figures and images for: Diurnal Variation in Systemic Acute Inflammation and Clinical Outcomes Following Severe Blunt Trauma
Source: Front Immunol. 2019 Nov 20;10:2699. doi: 10.3389/fimmu.2019.02699 (PMC6879654; doi:10.3389/fimmu.2019.02699)

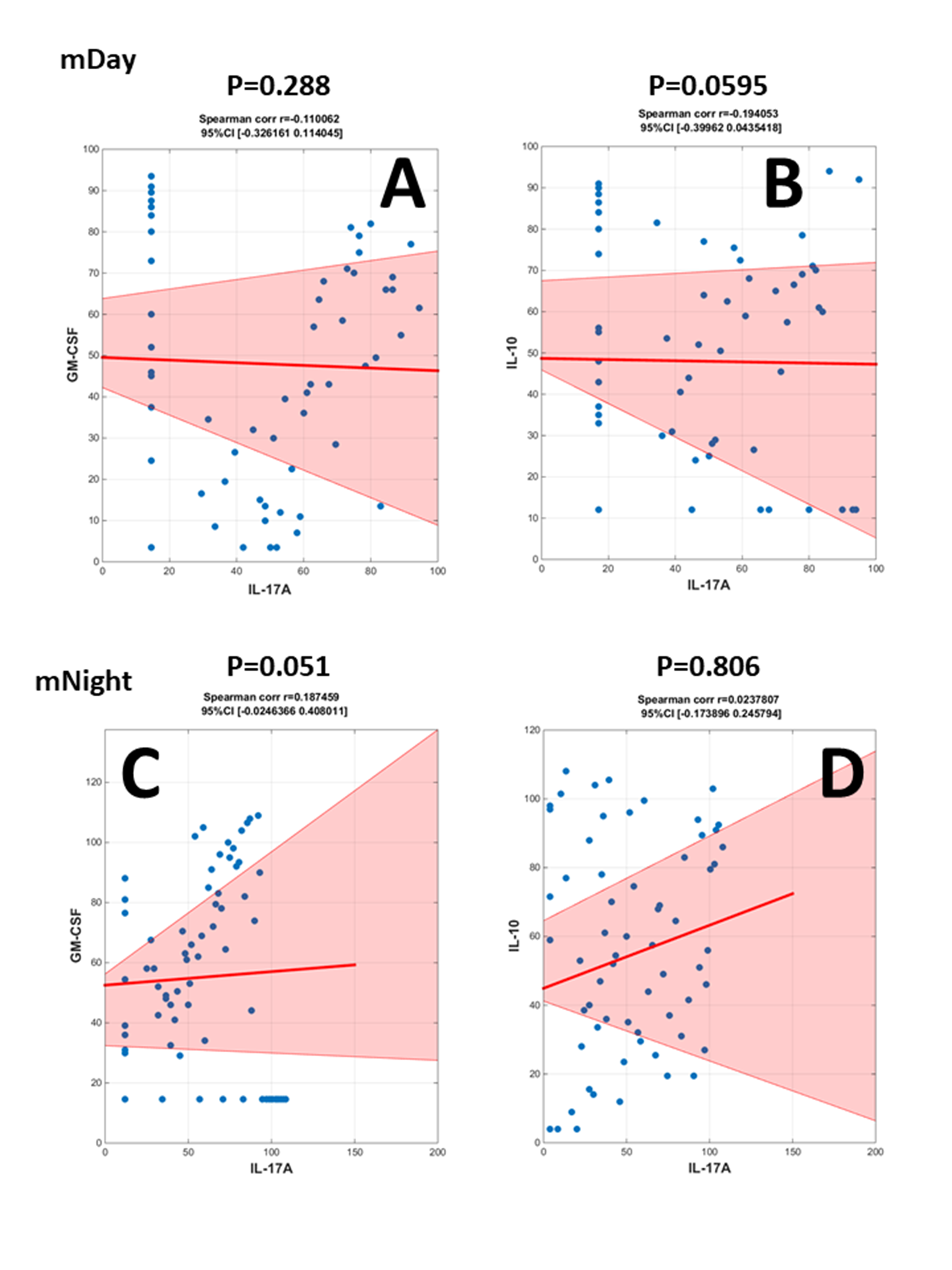

Supplement: Supplementary Figure 1 — Correlation analyses aimed at defining Th17 cell subsets computationally. Spearman correlations were carried out on mDay vs. mNight patient cytokine data to suggest the presence of pathogenic (IL-17A vs. GM-CSF; A,C) and non-pathogenic (IL-17A vs. IL-10; B,D) Th17 cells. This analysis suggested the possible predominance of pathogenic Th17 cells in the mNight group and non-pathogenic Th17 cells in the mDay. [file Image_1.TIF]
